# Somatisation Scores Associated With Healthcare Costs and Utilisation in a Sample of Emergency Department Patients

**DOI:** 10.1111/1742-6723.70246

**Published:** 2026-03-31

**Authors:** Ben Britton, Ria Mittal, Keira Barnard, Samantha Chapman, Damian Chen, Benjamin De Berg, Vinodkumar Raveendran, Elizabeth Pepper

**Affiliations:** ^1^ University of Newcastle Newcastle New South Wales Australia; ^2^ Hunter New England Health New Lambton Heights New South Wales Australia

**Keywords:** Australian, healthcare utilisation, public hospital, retrospective cohort study, somatic symptom

## Abstract

**Objectives:**

Somatic Symptom and Related Disorders (SSRD) are associated with frequent healthcare utilisation and elevated costs, yet their impact within Emergency Departments (EDs) remains underexplored. This study investigates the relationship between somatisation score and healthcare utilisation and costs in an Australian ED setting.

**Design:**

Retrospective cohort study.

**Setting:**

A tertiary public hospital ED in Australia serving metropolitan and rural populations. Healthcare utilisation and cost data were extracted from the local network's Activity Based Management database for 3.5 financial years preceding the index presentation.

**Participants:**

375 ED patients aged 18–70 years screened using the PHQ‐15 and WI‐7 tools over a two‐week period. Participants were classified as high or low somatisers using dynamic cutoffs. Total available healthcare costs and occasions of service over the 42 months preceding the index ED presentation were gathered. Negative binomial regression models were used to identify predictors of total costs and service occasions.

**Results:**

High somatisers (59.84% of the sample) incurred significantly greater healthcare costs (mean AUD $23,713 vs. $10,392) and service occasions (mean 38.7 vs. 18.7) than low somatisers. regression analyses identified somatisation severity, age and female sex as significant predictors of increased healthcare utilisation and costs. Presentation‐based variables such as triage category and diagnosis were not significant predictors.

**Conclusions:**

High somatisation scores are strongly associated with increased healthcare utilisation and costs in ED patients. These findings suggest that SSRD may be a previously neglected factor in hospital resource planning. Early identification and appropriate management of SSRD in EDs could yield substantial economic and clinical benefits.

## 1 Introduction

Somatic Symptom and Related Disorders (SSRD) are a group of psychiatric conditions characterised by persistent, distressing physical symptoms accompanied by excessive thoughts, feelings, or behaviours related to these symptoms, leading to significant functional impairment [1]. The category includes Somatic symptom disorder, conversion or functional neurological disorder, illness anxiety disorder and factitious disorder.

Individuals with SSRD often seek frequent medical attention for symptoms not fully explained by medical assessment, potentially leading to unnecessary tests, treatments, iatrogenic harm and patient frustration [2, 3]. This, combined with associated psychiatric comorbidities, contributes to substantial personal and economic burdens, including lost productivity [3].

While the impacts of these illnesses on the patient are great, there are also implications for the hospital systems providing the care. Operational bottlenecks in Emergency Departments (EDs) and in particular ED overcrowding and extended length of stays are inextricably linked with whole‐hospital inefficiencies [4]. It has been estimated that patients who stay more than 1 day in ED have an 11.7% increase in hospital length of stay [4]. The ability to identify patients that may contribute to ED inefficiencies and therefore overall hospital costs is widely desired by hospital administrations. Although anecdotally implicated in ED costs, one area of ED inefficiency that has not been closely studied is SSRDs.

In areas outside the ED, there is consistent evidence that individuals with higher somatic symptom severity disproportionately use healthcare services. Studies in primary care report differences in healthcare utilisation and costs of mean $2734 and €5288 [5] greater over 12 months for those with high somatic symptoms. In outpatient settings, the difference was $358 with 2.6 more visits [6]. An Australian study on surgical inpatients found 52% of their sample to be high somatisers and a mean difference of €1401 in healthcare utilisation between high and low somatization groups [7]. Overall, individuals with higher scores on somatization screening tools have shown up to nine times higher healthcare costs than the general population in some UK studies [8]. A New Zealand study found a median cost of £1221 for patients with medically unexplained symptoms, but lacked a control group [9]. Despite this wide range of settings, to date there has only been limited research on healthcare utilisation and costs for patients presenting to EDs.

Higher costs may be avoidable. The initial conversation providing SSRD diagnosis itself has therapeutic value [10]. In a trial with patients with Dissociative Seizures, a subset of SSRD, high‐quality communication about their diagnosis reduced healthcare costs by −66.2% per subsequent year, while poorly communicated diagnoses led to an increase of 363.6% [11].

Once an accurate and well‐communicated diagnosis has been provided, there are effective treatments for people with SSRD. Effective management most often involves integrating psychological therapies such as Cognitive Behaviour Therapy and sometimes pharmacological treatments [10, 12]. When appropriately provided, treatment for SSRD also has economic impacts, reducing healthcare costs in those treated in one study by 24.5% over 2 years [13] and significantly reducing ED visits in another [6].

Despite frequent patient presentations, potentially high costs and the unrealised potential for intervention in this setting, SSRD is not well researched in EDs, resulting in limited resources, training and management protocols. This project aimed to examine the severity of somatic symptoms and associated healthcare utilisation and costs of people presenting to an Australian emergency department.

We hypothesised that patients categorised as ‘high somatiser’ based on validated questionnaires will demonstrate significantly greater total healthcare costs and service utilisation over the previous 42 months (June 2019–December 2022) compared to those categorised as ‘low somatiser’.

## 2 Method

### 2.1 Design and Setting

This project was a retrospective cohort design. It was a collaboration between the Adult Consultation Liaison Psychiatry and Neurology departments of a local public tertiary hospital in Australia. Supervised medical students from a local university assisted in screening for somatic symptom disorder in the ED. The local ED manages approximately 15,000 presentations annually [14] and serves a broad population living in both metropolitan and rural areas.

### 2.2 Ethics

Ethical approval for the study was granted by the local Human Research Ethics Committee on May 18, 2023 (HREC 2023/ETH00645).

### 2.3 Participants

Study participants were ED patients aged 18–70 years, presenting between 10 AM and 10 PM during a 2‐week period from June 12–25, 2023. Incomplete surveys were excluded, resulting in a final sample size of 375 participants.

#### 2.3.1 Exclusions

1. Patients younger than 18 [15] or older than 70 years, as the screening tools are not validated for children and because older patients have a higher prevalence of multimorbidity and cognitive impairment affecting tool validity [16].
2. Patients requiring ongoing resuscitation or deemed too unstable by the treating medical officer.
3. Patients presenting with a reduced level of consciousness, delirium, other cognitive impairment, or exhibiting aggressive or agitated behaviours, as identified by the treating medical officer.

### 2.4 Procedure

Student researchers identified eligible individuals via ED patient lists and consulted with their treating team for suitability before approaching and inviting them to participate.

Informed consent to participate and questionnaire data were collected via REDCap [17] a secure online web survey hosted by the local health district. Paper versions were provided if digital completion was not possible.

For each participant, healthcare utilisation and cost data were extracted from the local health network's Activity Based Management (ABM) database for the period of June 2019 to December 2022. This 42‐month retrospective window represents the most recent finalised financial data available at the time of recruitment in June 2023, accounting for the standard 6‐month administrative lag in the ABM reporting system.

### 2.5 Measures

The ABM database provides the health service with financial and service utilisation data on clinical encounters and is used to inform resource allocation. In this study, data were collected on 75 variables, including age, Indigenous status, sex, ED triage category, ED diagnosis, discharge specialty, bed days, ED and inpatient average length of stay (ALOS), psychiatric inpatient days and Intensive Care Unit (ICU) hours. These data were used to calculate total costs and occasions of service, with the latter defined as discrete clinical encounters recorded within the health district. This includes, but is not limited to, contacts related to allied health, critical care, medical specialty consultations, diagnostic imaging, operating room procedures and pathology services.

The Patient Health Questionnaire‐15 (PHQ‐15) [12] is a widely used, reliable and validated 15‐item self‐report instrument for somatic symptom severity. It assesses 15 common somatic symptoms and how often respondents have been bothered by them over the past 4 weeks on a two point scale (0 ‘not bothered at all’ to 2 ‘bothered a lot’), yielding a total score from 0 to 30. Higher PHQ‐15 scores have been associated with somatisation independent of other comorbid physical or psychiatric complaints [3] and the PHQ‐15 has been validated as a screener for SSRD [18].

The Whiteley Index‐7 (WI‐7) is a brief, seven‐question adaptation validated for somatoform disorders, primarily in primary care settings [19]. It evaluates illness concerns and health conviction. Respondents answer ‘yes’ or ‘no’, with scores from 0 ‘minimal concern’ to 7 ‘high preoccupation’. It was selected for this study primarily due to its clinical utility in acute settings like the Emergency Department. While longer indices like the WI‐14 or WI‐28 exist, the 7‐item version has been specifically validated as an efficient screening tool for somatoform disorders in medical settings. Most importantly, the use of the WI‐7 was dictated by the ‘dynamic cutoff’ protocol [20], which was utilised to improve diagnostic accuracy by combining somatic symptom severity (PHQ‐15) with health preoccupation (WI‐7). This approach allows for a more nuanced classification of ‘high somatisers’ than using either scale in isolation.

Categorising ‘high somatisers’ was done using the previously published protocol combining screening for the somatic (PHQ‐15) and the psychological (WI‐7) characteristics of SSRD to provide more accurate and efficient diagnoses [20]. A series of dynamic cut‐off scores has been developed where the threshold that identifies a ‘high somatiser’ on the WI‐7 changes depending on the score of the PHQ‐15 and vice versa. As the PHQ‐15 score increases, indicating higher symptom severity, the corresponding WI‐7 cut‐off score necessary for categorisation decreases. These dynamic cut‐offs provide a sensitivity and specificity of 0.73 and 0.77 respectively [20] (see Box 1 for details).

BOX 1 **Dynamic Cut‐Offs for Diagnosis of SSRD. Based on Laferton et al. [20].**

**Table:** 

PHQ‐15	0	1	2	3	4	5	6	7	8	9	10	11	12	≥13
WI‐7	4	4	3	3	3	3	2	2	2	2	1	1	1	0

### 2.6 Statistical Analysis

Analyses were conducted using STATA 18 [21]. Demographics were evaluated using *t*‐tests for continuous variables and chi‐square tests for categorical variables. Due to significant overdispersion in the healthcare cost and utilisation data (where variance substantially exceeded the mean), negative binomial regression models were employed as they are better suited for skewed count and cost data than Poisson models. The exploratory regressions began with saturated models containing all potential predictors of cost and occasions of service: age, sex (defined as a dichotomous variable from medical records), Indigenous status, ED triage category, ED diagnosis, principal procedure and discharge specialty. A manual backward elimination approach was employed to identify the most parsimonious models for each outcome. Variables were removed one at a time, starting with the highest non‐significant *p*‐value, until all remaining predictors reached a significance level of < 0.05. Model fit was compared at each step using the Likelihood Ratio test to ensure the removal of variables did not significantly degrade the model's explanatory power.

## 3 Results

Of the 375 participants, 225 (59.84%) were identified as high somatisers using the dynamic cut‐offs.

Demographic analysis (Table 1) revealed significant differences between High and low somatisers. Females were significantly more likely to score as high somatisers. There were also differences in triage category and presenting complaints.

**TABLE 1:** Demographics.

SSD using dynamic cutoffs	High somatisers	Low somatisers
	59.84% (225)	40.16% (151)
Mean age (SD)	38.88 (16.40)	41.95 (15.49)
Female (*n*)**	62.50% (140)	43.7% (66)
Aboriginal and not Torres Strait Islander	11.45% (19)	4.59% (5)
Triage category		
Resuscitation^a^ (1)	0.45% (1)	0% (0)
Emergency (2)	20.98% (47)	16.56% (25)
Urgent (3)	35.71% (80)	25.83% (39)
Semi‐Urgent (4)	41.07% (92)	49.01% (74)
Non‐Urgent (5)	1.79% (4)	8.61% (13)
Presenting complaint		
Dizziness	0.45% (1)	0% (0)
Infection	13.84% (31)	7.28% (11)
Other	16.96% (38)	15.89% (24)
Pain	50.00% (112)	33.11% (50)
Psychiatric	1.34% (3)	0% (0)
Shortness of breath	5.80% (13)	2.65% (4)
Trauma	11.61% (26)	41.06% (62)

^a^ Stabilised by time of recruitment.

** Significant at 0.001.

In the 42 months preceding the index presentation, high somatisers incurred costs on average of $13,000 more than low somatisers (Table 2).

**TABLE 2:** Differences in costs (AUD) and occasions of service in high and low somatisers.

	Total	Mean	(SD)	Median	(25th–75th %)
Total cost					
Low somatisers (*n* = 151)	$1,569,342	$10,392	(30,900)	$850	(0–6455)
High somatisers (*n* = 225)	$5,335,511	$23,713	(64,607)	$2763	(0–13,621)
Total occasions of service					
Low somatisers (*n* = 151)	2826	18.72	(106.86)	2	(1–8)
High somatisers (*n* = 225)	8708	38.70	(126.72)	5	(1–20)

The mean number of healthcare occasions of service for high somatisers was 20 more than low somatisers (Table 2).

In negative binomial regression models (Table 3), high somatisation and age were significant predictors of total costs and occasions of service. In addition, being female was also a significant predictor of occasions of service. Sex, indigenous status, ED triage category, ED diagnosis, presenting complaint, principal procedure and discharge specialty did not significantly contribute to the models and were removed.

**TABLE 3:** Most parsimonious negative binomial models of total costs and occasions of service over the period.

Variable	Binomial coefficient	*p*	95% CI
Model of predictors of total costs			
High somatiser	0.90	0.002	0.35–1.45
Age	0.02	0.034	0.00–0.04
Model of predictors of occasions of service			
High somatiser	0.97	< 0.001	0.62–1.32
Age	0.03	< 0.001	0.02–0.04
Female	0.60	0.001	0.25–0.96

## 4 Discussion

This study was the first of its kind to examine healthcare utilisation related to somatisation scores of patients presenting to an Australian Emergency Department.

Of the 375 patients recruited, 59.84% scored as high somatisers. While this prevalence is high, it is in keeping with other studies of SSRD in health settings [7] and specifically in EDs [22, 23], supporting the assertion that SSRD are an area requiring attention.

The results reported here demonstrated that as hypothesised, High Somatisation strongly predicted greater healthcare utilisation and costs. These findings supported existing literature showing increased healthcare costs and service utilisation for those with higher somatisation scores in other settings [3, 7]. Age was also a significant independent predictor, with older people costing more and receiving a significantly greater number of occasions of service for each additional year of life. Finally, women had significantly more occasions of service. These patterns of healthcare utilisation increasing with age and weighted towards women are mirrored in Australian and overseas literature [24, 25].

The results of this study are also notable for what was *not* associated with greater healthcare utilisation and costs in a model with somatisation.

Presentation‐based predictors such as ED Triage Category, ED diagnosis, presenting complaint, principal procedure and discharge specialty did not significantly predict healthcare utilisation and costs in the model. In this study, somatisation was a stronger predictor than any of these factors of who was a high‐cost, high‐utiliser of health care. The implication of these findings is that prioritising the identification and management of somatisation in the ED may contribute to greater hospital efficiency and better value healthcare.

A notable finding was the differing clinical profiles between groups: the low somatisation group had a higher proportion of trauma presentations, while the high somatisation group was characterised by a higher prevalence of pain‐related complaints. While our models suggest somatisation was an independent predictor, the inherent cost differences between managing acute trauma and non‐specific pain presentations should be considered in future, larger‐scale replications.

Indigenous status was not a statistically significant predictor of healthcare utilisation or cost. However, this result must be interpreted with caution due to the small sample size (*n* = 24 of 375 participants). This aligns with research that found no difference between Indigenous and non‐Indigenous Australians when other health factors were considered [26]. This study provides the suggestion that one such health factor to control for should be somatisation, as of the 24 participants identified as Aboriginal, a significant number (*n* = 19, 79%) scored as high somatisers. There is currently no literature on the prevalence of somatisation in Aboriginal Australians to guide the discourse. Whether Aboriginal people are more likely to be high somatisers or whether due to cultural differences they only present as such on scales developed in Western cultures is beyond the scope of this study, but further research in this area is clearly needed.

There were several strengths of the study including the relatively open inclusion criteria and ecological validity of recruiting directly from ED. SSRD is under‐investigated in emergency medicine literature, limiting management protocols and staff training, despite frequent patient presentations. This study attempts to address that gap in knowledge. Methodological strengths were the objectivity of the healthcare utilisation data and that its collection was blinded to somatisation score.

Collecting data in the ED as a convenience sample was a limitation of the study. Medical students approached all accessible patients, but some were excluded due to being too unstable or inappropriate for screening at the time. Collected this way, information like medical complexity was not available. Because ethics protocols did not allow for retention of data on individuals who declined or did not complete surveys, we lack information on the clinical profiles or medical complexity of those excluded. This may have led to a selection bias; specifically, individuals with higher levels of somatic preoccupation may have been more motivated to engage in a study focused on their symptoms, potentially contributing to the high proportion (60%) of high somatisers in this sample. Consequently, the sample may not be fully representative of the general ED population.

Another limitation of the study was that scores on the Whiteley Index can be influenced by sex, with some literature suggesting females may score higher on somatisation symptoms. As females made up 55% of the ED sample, it is possible that this over‐inflated the prevalence of somatisation due to biased instruments. While our regression model identified female sex as an independent predictor of occasions of service over and above somatisation, the potential for collinearity between sex and somatisation scores must be considered when interpreting these results.

The inclusion criteria, despite being broad, were limited to 18–70 year old participants due to the decreasing validity of the scale for people over 70. Not being able to include the elderly was a significant limitation of the study and limits the generalisability of the findings.

Another limitation of the study was a result of its novel nature. While the somatisation scales are well validated in Primary Care settings, only once have they been used in an Emergency Department and never in Australia [27]. It's possible that the different medical and cultural settings of an Australian ED may impact on the validity of the scales and not accurately estimate the prevalence of somatisation.

A final limitation of our study and others like it is that a retrospective cohort design does not allow causal inferences to be made. Whether high somatisation caused increased healthcare costs and utilisation is not definite and the inverse must be considered: that people with high healthcare utilisation become high somatisers.

An additional consideration of this study was that the three and a half‐year data collection period coincided with the COVID‐19 pandemic, which imposed significant biopsychosocial, economic, social and financial burdens, particularly on primary care and EDs. Studies indicate that individuals with SSRD likely experienced increased symptom burden during the pandemic due to psychosocial impacts [28].

The absolute financial difference of a sample of 2 weeks of ED patients of $3.7 million over 42 months highlights a substantial economic burden, prompting discussions on optimising resource allocation. The potential savings generated by addressing somatisation would be large and self‐funding.

There are several ways in which somatisation may be addressed in EDs. Addressing the lack of knowledge and confidence in working with people with FND reported by ED clinicians [29, 30], education about the condition is an important first step. The introduction of screening for somatisation in ED presenters could help identify SSRD and trigger an appropriate referral to address it. Once identified, training in communicating the diagnosis well [31, 32] and early [31, 33] improves outcomes. Further intervention with evidence‐based, multidisciplinary treatment, integrating psychotherapy, pharmacotherapy and allied health services has been shown to benefit people with SSRD [6, 10, 31, 33]. It is important to note that people with SSRD can have other comorbid health complaints. As such, while people with SSRD may benefit from targeted interventions, these should be designed to complement and not replace standard medical care provided in the ED.

## 5 Conclusion

This study found that high somatic symptom severity is associated with significantly higher healthcare utilisation and costs. These findings suggest that SSRD may be prevalent in the emergency department, warranting its consideration in clinical assessment and case formulation. Further, the scale of the costs associated with high somatisation suggests that interventions to address it would be self‐funding and still produce net savings.

Our results highlight a significant opportunity to improve recognition and management of patients with SSRD in the ED. Future research should explore optimal identification and intervention strategies with the aim of reducing healthcare utilisation and costs, improving hospital efficiencies and operations and ultimately to improve the lives of people living with SSRD.

## Author Contributions

All authors of this paper meet the ICMJE criteria for authorship. **Ben Britton:** conceptualisation, methodology, formal analysis, investigation, data curation, writing the original draft, review and editing, supervision and project administration. **Ria Mittal:** conceptualisation, methodology, formal analysis, investigation, data curation, writing the original draft, review and editing and project administration. **Keira Barnard:** conceptualisation, methodology, formal analysis, investigation, data curation, review and editing and project administration. **Samantha Chapman:** data curation and project administration. **Damian Chen:** data curation and project administration. **Benjamin De Berg:** data curation and project administration. **Vinodkumar Raveendran:** conceptualisation, methodology, investigation, data curation, review and editing, supervision and project administration. **Elizabeth Pepper:** conceptualisation, methodology, investigation, data curation, writing the original draft, review and editing, supervision and project administration.

## Funding

The authors have nothing to report.

## Conflicts of Interest

The authors declare no conflicts of interest.

## Data Availability

The data that support the findings of this study are available on request from the corresponding author. The data are not publicly available due to privacy or ethical restrictions.
